# The Zebrafish Mutants *dre, uki,* and *lep* Encode Negative Regulators of the Hedgehog Signaling Pathway 

**DOI:** 10.1371/journal.pgen.0010019

**Published:** 2005-08-19

**Authors:** Marco J Koudijs, Marjo J. den Broeder, Astrid Keijser, Erno Wienholds, Saskia Houwing, Ellen M. H. C. van Rooijen, Robert Geisler, Fredericus J. M. van Eeden

**Affiliations:** 1 Hubrecht Laboratory, The Netherlands Institute for Developmental Biology, Utrecht, The Netherlands; 2 Max-Planck-Institut für Entwicklungsbiologie, Tübingen, Germany; University of Pennsylvania School of Medicine, United States of America

## Abstract

Proliferation is one of the basic processes that control embryogenesis. To identify factors involved in the regulation of proliferation, we performed a zebrafish genetic screen in which we used proliferating cell nuclear antigen (PCNA) expression as a readout. Two mutants, *hu418B* and *hu540A,* show increased PCNA expression. Morphologically both mutants resembled the *dre (dreumes), uki (ukkie),* and *lep (leprechaun)* mutant class and both are shown to be additional *uki* alleles. Surprisingly, although an increased size is detected of multiple structures in these mutant embryos, adults become dwarfs. We show that these mutations disrupt repressors of the Hedgehog (Hh) signaling pathway. The *dre, uki,* and *lep* loci encode Su(fu) (suppressor of fused), Hip (Hedgehog interacting protein), and Ptc2 (Patched2) proteins, respectively. This class of mutants is therefore unique compared to previously described Hh mutants from zebrafish genetic screens, which mainly show loss of Hh signaling. Furthermore, *su(fu)* and *ptc2* mutants have not been described in vertebrate model systems before. Inhibiting Hh activity by cyclopamine rescues *uki* and *lep* mutants and confirms the overactivation of the Hh signaling pathway in these mutants. Triple *uki/dre/lep* mutants show neither an additive increase in PCNA expression nor enhanced embryonic phenotypes, suggesting that other negative regulators, possibly Ptc1, prevent further activation of the Hh signaling pathway. The effects of increased Hh signaling resulting from the genetic alterations in the *uki, dre,* and *lep* mutants differ from phenotypes described as a result of Hh overexpression and therefore provide additional insight into the role of Hh signaling during vertebrate development.

## Synopsis

In a screen aimed at finding genes that control proliferation in the zebrafish embryo, three mutants were identified. Mutants showed an increase in size of several structures including the brain, the retina, and the fins. Surprisingly, although size was increased in the embryos, adults remained small. Cloning of these genes revealed that increased Hedgehog signaling was at the basis of the phenotype, because all three genes encoded known repressors of the Hedgehog signaling pathway: Ptc2, Su(Fu), and Hip.

Hedgehog is known to play a role in proliferation. For instance, ectopic Hedgehog signaling can lead to several tumors including basal cell carcinoma and medulloblastoma. However, the phenotypes were still a surprise, because earlier experiments in zebrafish embryos suggested that activation should lead to patterning rather than proliferation defects. Current models of the pathway predict that these genes act independently to inhibit the signal but curiously, redundancy amongst these genes was not found, because triple mutants looked like the single mutants.

The conclusion is that weak activation of Hedgehog signaling can already lead to stimulation of growth in the absence of patterning defects, and that the Hedgehog signal is probably kept in check by the last inhibitor: Ptc1. A mutant for the *ptc1* gene has recently been created and will put the model to the test.

## Introduction

During development, proliferation is one of the key processes in the formation of an embryo, but how it is controlled spatiotemporally is still poorly understood. A tight regulation of proliferation is necessary during development and the remaining lifespan of an organism, as disrupted regulation might result in tumorigenesis. Several essential developmental signaling pathways are reported to control embryogenesis and many of these are involved in regulating proliferation in vertebrates and invertebrates. These basic developmental pathways all involve receptor ligation of highly conserved sets of secreted peptides like the TGF-β superfamily [1], FGF [2], Wnt [3], and Hedgehog (Hh) [4]. The Hh signaling pathway is highly conserved throughout evolution and has been documented to control proliferation [5]. In our current understanding, Hh proteins are expressed in a signaling cell, secreted and bound to the 12-transmembrane receptor Ptc (Patched) on a neighboring cell. Upon this binding, Ptc is thought to be internalized into endosomes where it is unable to repress the activity of Smo (smoothened) [6,7]. The signal is transmitted to the downstream proteins Cos2 (Costal2), Fused, Su(fu) (suppressor of fused), and one of the at least three members of the Gli family of zinc finger transcription factors [4]. In the presence of Hh, the Gli protein can be activated and transported to the nucleus where it activates genes mainly involved in patterning, proliferation, and cell structure [8]. Multiple genes are described to limit the activity of Hh signaling. Besides its own receptors Ptc1 and Ptc2, Hip (Hedgehog interacting protein) [9] is also expressed at the membrane in response to Hh activity. All three are involved in sequestering Hh to limit its effective range [10]. Further down the pathway, casein kinase I (CKI), glycogen synthase 3β (GSK3β), and protein kinase A (PKA) are involved in the processing or degradation of the Gli transcription factor [4]. The nuclear activity of Gli proteins is inhibited by Cos2 (Costal2) [11–13] and Su(fu) [14–18], which are both reported to be involved in tethering Gli in the cytoplasm, preventing overactivation of the pathway.

Hh signaling regulates multiple developmental processes in specific tissues in vertebrates and invertebrates [4]. In addition to the role of Hh during development, it is necessary to tightly regulate its activity during adulthood, where its aberrant activation is reported to predispose to malignant types of tumors in bone [19], pancreas [20], gut [21], skin, and brain [22,23]. Mutations in the negative regulator Su(fu) have been reported to predispose to medulloblastomas [24]. The formation of medulloblastomas has also been observed in patients suffering from Nevoid basal-cell carcinoma syndrome (NBCCS), where mutations in the Ptc protein have been identified [25].

Here we report about a forward genetic screen, performed to identify proliferation mutants. In this screen we used an in situ hybridization (ISH) approach in which we used PCNA expression levels as a specific marker for proliferation. Two zebrafish mutants, called *hu418B* and *hu540A,* were identified showing increased levels of proliferation at 40 h post fertilization (hpf). After 4 d, a combination of phenotypes was observed, similar to a known class of mutants from the Tübingen large-scale zebrafish screen [26]. These mutants, known as *dre (dreumes), uki (ukkie),* and *lep (leprechaun),* were identified based on their eye [27], ear [28], and pectoral fin [29] phenotypes. Both proliferation mutants from our screen are shown to be additional alleles of the *uki* mutation. In addition to the previously described phenotypes, adult *dre* mutants specifically show a disturbed regulation of chondrocyte differentiation in the branchial arches.

Positional cloning of this class of mutants identified mutations in negative regulators of Hh signaling. The *dre, uki,* and *lep* mutants encode the zebrafish homologs of the negative regulators Su(fu) [30], Hip [9], and Ptc2 [31]. As a result, the Hh signaling pathway is aberrantly activated. Treating mutant embryos with cyclopamine, a specific inhibitor of Hh signaling, rescues the phenotypes of *uki* and *lep* mutants. In an attempt to enhance the level of proliferation, double and triple mutants were generated showing equal levels of proliferation, compared to *uki^hu418B^* and *uki^hu540A^,* and no additive effect on the embryonic phenotypes. This suggests that additional regulators are still capable of inhibiting the Hh pathway, preventing further activation. In this report we describe the identification of the first vertebrate *su(fu)* and *ptc2* mutants, and three nonsense mutations in *Hip,* all showing similar phenotypes. All mutants demonstrate the effects of aberrant activation of Hh signaling, which differs from all previously described Hh mutants in the zebrafish, which mainly show inhibition of Hh activity. This class of mutants will therefore contribute to the understanding of the role of Hh signaling during vertebrate development.

## Results

### A Genetic Screen for Proliferation Mutants

**Figure 1:**
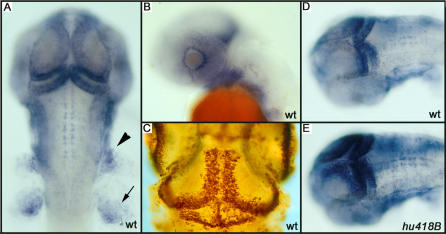
PCNA and Proliferation Patterns (A) PCNA pattern as scored during the screen. In a dorsal view at 40 hpf, PCNA staining is observed in the medial and posterior part of the tectum, and in the cerebellum, the neural crest (arrowhead), and the pectoral fin (arrow). (B) In a sideview (42 hpf), a ring of positive cells is visible around the lens. (C) In a whole-mount BrdU labeling from day 3.5 to 4.5, similar regions are labeled indicating that PCNA RNA expression prefigures where BrdU will be incorporated. (D and E) Sibling and *hu418B* mutants, respectively, showing increased PCNA labeling in the CMZ, but most prominently in the tectum.

To identify mutants showing altered levels of embryonic proliferation, we used proliferating cell nuclear antigen (PCNA) as a readout, a commonly used marker in proliferation studies. PCNA is a protein that cooperates with DNA polymerase δ during DNA replication and repair [32]. We found that it was difficult to use the standard antibody (PC10, Novocastra Laboratories Ltd, Newcastle upon Tyne, United Kingdom) in a whole-mount procedure, but PCNA ISH gave robust results. Early in development, all cells are PCNA positive and expression gradually diminishes as patterning and differentiation proceed. At 40 hpf, a number of tissues are still positive, and these correspond to ones known to develop late, e.g., the pectoral fins, the gut, and the branchial arches. Furthermore, the cells that line the lumen of the neural tube, or cells in several brain folds that are derived from that region, are PCNA positive (Figure 1A). Finally, a ring of cells around the lens, called the ciliary marginal zone (CMZ), is PCNA positive (Figure 1B). The CMZ is known to contain stem cells that continue to generate retinal cells throughout life in lower vertebrates [33]. BrdU (bromo-2-deoxy-uridine) labeling experiments have shown that these regions indeed contain actively dividing cells (Figure 1C) [34]. Later in development, expression further decreases and at 120 hpf, PCNA expression becomes almost undetectable.

We screened 100 mutagenized genomes for mutations that affect the level of PCNA expression. Several mutants with an increased expression were noted, but the majority showed typical degeneration/apoptosis phenotypes. Two mutants, however, showed an increase in PCNA expression, most prominently visible in the tectum, and did not show degeneration**.** These mutants, named *hu418B* and *hu540A,* show an increased expression of PCNA in the peripheral retina and the tectum (Figure 1D and 1E). These regions are known to be highly proliferative and are thought to contain stem cells. The increase can be observed at 36 hpf, but not at 24 hpf when a larger proportion of the embryo is PCNA positive. Furthermore, expression still undergoes a general reduction as the embryo ages. At 96 hpf, PCNA is difficult to detect in both mutant and wild-type embryos using an ISH approach. To exclude the possibility that a low level of apoptotic cell death is responsible for the increase of PCNA expression, we performed a whole-mount terminal transferase dUTP nick-end labeling (TUNEL) assay on *hu418B* mutants and siblings at 40 hpf. No difference in the level of apoptosis could be observed (data not shown), indicating that these mutants purely display increased levels of PCNA, independent of apoptosis.

### Altered Level of Proliferation Affects Several Structures of the Developing Embryo

**Figure 2:**
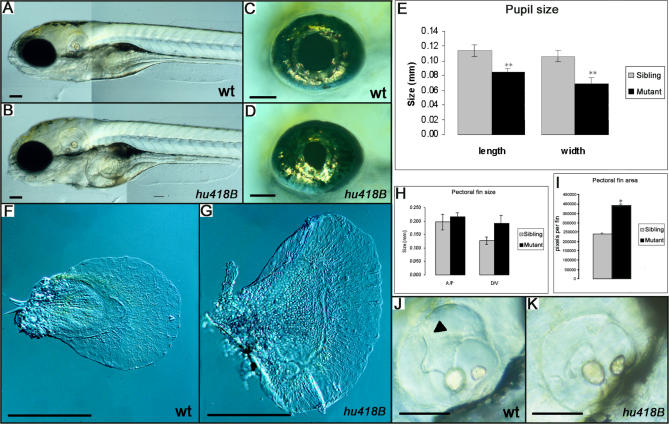
Phenotypes of *uki^hu418B^* Mutant Embryos (A and B) Lateral view of a wild-type (wt) and *uki^hu418B^* mutant. (B) Showing an increased volume of the head. The size of the pupil is reduced in the *uki^hu18B^* mutant without affecting the size of the eye. (C–E) The size of the pupil is reduced in the *uki* ^hu18B^ mutant compared to wild-type, without affecting the size of the eye. Measurements revealed that the length and width of the pupil is significantly reduced in the *uki^418B^* mutant (*n* = 5, *p < 0,001). (F–H) Pectoral fins showing the increased size of an *uki^418B^* mutant in which the dorsal/ventral (D/V) size of the pectoral fin is increased by 50%, (*n* = 3, *0.01 < *p* < 0.02). (I) The anterior/posterior size is not significantly affected, but the fin area has increased by 65% (*n* = 3, *p* < 0.001). (J and K) *uki^hu418B^* mutants lack the dorsolateral septum in the ear (arrowhead). Scale bar is 100 μm.

Morphological analysis of *hu418B* and *hu540A* mutants shows abnormalities that correspond with an increased level of cell proliferation. At 72 hpf, the volume of the head is increased (Figure 2A and 2B), mainly in the region of the tectum, where an increased level of proliferation is observed. Additionally, *hu418B* and *hu540A* mutant embryos show a reduced size of the pupil, whereas the overall size of the eye is unaffected (Figure 2C and 2D). Measurements revealed that both length and width of the pupil are significantly decreased (Figure 2E). However, the formation of the lens is normal (data not shown). This eye phenotype might result from the increased rate of proliferation in the CMZ. The retina is reported to grow as a result of several division steps of retinal stem cells in the CMZ [35]. An increased level of proliferation of these cells might cause the retina to overgrow the lens, reducing the size of the pupil. Additionally, the pectoral fins of the *hu418B* and *hu540A* mutants were enlarged (Figure 2F and 2G). Dorsoventrally the fins have approximately increased in size by 50% (0.01 < *p* < 0.02, *n* = 6), the area has increased by 65% (*p* < 0.001, *n* = 3) (Figure 2H and 2I).

An additional phenotype is observed in the otic vesicle in the *hu418B* and *hu540A* mutants, in which the dorsolateral septum is missing (Figure 2J and 2K). However, the otoliths are correctly positioned, indicating that the anlage of the ear is correct.

This phenotypic combination was already observed in a class of mutants identified in a large-scale screen [26], covering the *dre, uki,* and *lep* mutants [27–29]. Complementation analysis revealed that *hu418B* and *hu540A* are additional alleles of *uki* (now referred to as *uki^hu418B^* and *uki^hu540A^*). Of this complementation group, the *uki^hu418B^* mutant shows the most consistent and strongest phenotype and is therefore used for further experiments. The morphological phenotype of *dre* and *lep* is slightly weaker and no clear difference in PCNA expression can be detected in the *dre* and *lep* mutants using an ISH approach.

### Adult Mutants Show Additional Phenotypes

Raising homozygous *uki, dre,* and *lep* mutants demonstrates that approximately 10% of the *uki* and *lep* mutants reach 2 mo of age, and all die before the third month. Only *dre* mutants can be raised in significant numbers (50%) for 3 mo, with a maximum lifespan of 9 mo (5%). All mutants stay infertile and show a dwarfism phenotype [29]. This could be a result of the absence of growth hormone, which is secreted by the pituitary gland. However, sectioning an adult *dre* mutant revealed the presence of a pituitary gland (data not shown). The adenohypophysis secretes multiple hormones that are reported to play a role in the development of a dwarfism phenotype [36]. However, ISH experiments show that expression levels of growth hormone (GH), proopiomelanocortin (POMC), prolactin (PRL) and thyroid stimulating hormone (TSH) are not altered in *dre* mutants (data not shown).

**Figure 3:**
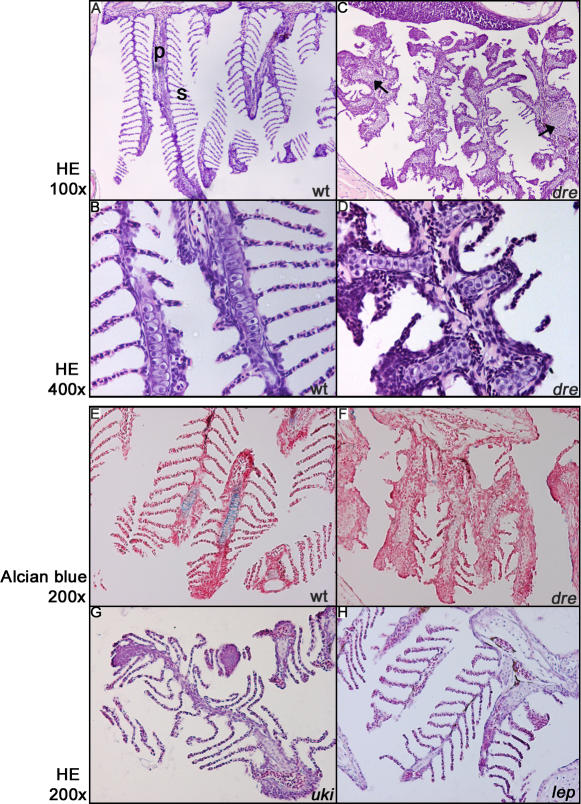
Patterning of the Branchial Arches in a 5-Mo-Old *dre* Mutant (A and B) The strict organization of the brachial arch into primary (p) and secondary (s) lamellae in a wild-type situation (100× magnification). Higher magnification shows stacks of single chondrocytes in the primary lamellae. (C and D) Sectioning of a *dre* mutant shows disturbed patterning, resulting in the absence of secondary lamellae and the presence of foci of chondrocyte-like cells in the primary lamellae (arrowsHE, hematoxylin and eosin stain; wt, wild-type. (E and F) Alcian Blue staining reveals the presence of differentiated chondrocytes in the wild-type (wt) primary lamellae, but not in the *dre* mutant, indicating that the differentiation of these chondrocytes is affected (200×). (G and H) Branchial arches of *uki* and *lep* mutants appear to be wild-type (wt).

Histological analysis of a 5-mo-old *dre* mutant (*n* = 4) and sibling (*n* = 3) reveals an additional phenotype concerning the gills of the adult fish. The gills contain branchial arches that are composed of multiple primary lamellae, formed by a stack of single chondrocytes (Figure 3A and 3B). To increase the area for sufficient oxygen uptake, these primary lamellae are branched into a large number of secondary lamellae (Figure 3A). However, in the *dre* mutant, the degree of branching to form secondary lamellae is severely diminished. The primary lamellae contain large clusters of cells, which morphologically resemble chondrocytes (Figure 3C). The strictly organized stacks of single chondrocytes are mainly absent. Occasionally, lines of chondrocytes appear to branch instead of the epithelium (Figure 3D). To investigate whether these clusters are indeed composed of chondrocytes we performed an Alcian Blue staining (Figure 3E and 3F) staining differentiated chondrocytes. In a wild-type fish the presence of differentiating chondrocytes could be confirmed in these single cell stacks. However, in the mutant, the clusters of cells were shown to be Alcian Blue negative (Figure 3F). On the other hand, the chondrocytes in the region where the primary lamellae are attached to the skeleton are Alcian Blue positive. This suggests that the chondrocytes in the gills are specifically affected in the *dre* mutant. One of the possibilities is that these Alcian Blue–negative cells are not able to properly differentiate during the development of the branchial arches. To examine whether these cells are in an earlier stage of cartilage formation, a Periodic Acid Schiff (PAS) staining was performed to detect ovotransferrin, a glycoprotein transiently expressed by differentiating hypertrophic chondrocytes, before they become Alcian Blue positive [37]. Both the mutant and the wild-type sections are negative for the PAS staining (data not shown). We therefore suggest that the chondrocyte-like cells in the mutant are prehypertrophic chondrocytes, which are arrested in their proliferative stage, which are therefore unable to finally differentiate into mature chondrocytes. This branchial arch phenotype appeared to be *dre* specific because primary lamellae in *uki* and *lep* mutants are able to branch (Figure 3G and 3H) and do not contain these foci of chondrocytes.

### The *dre* Locus is Encoding the Suppressor of Fused Protein

**Figure 4:**
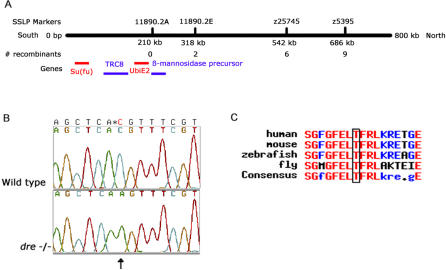
Positional Cloning of the *dre* Mutant (A) Schematic representation of assembled contig 11890 of the Zv2 genome assembly. SSLP markers z5395 and z25745 and newly identified SSLPs 11890.2A and 11890.2 were closely linked with the *dre* locus. Remaining recombinants of a complete panel of 765 mutant embryos are indicated. Four genes were predicted in the region of marker 11890.2A that encode Su(fu), TRC8, ubiquitin conjugating enzyme E2, and β-mannosidase precursor protein. (B) The *dre* mutation is a C to an A substitution, changing a threonine to a lysine. (C) Multiple alignment of Su(fu) homologs revealed that the induced mutation changes an amino acid in a highly conserved region of Su(fu).

**Figure 5:**
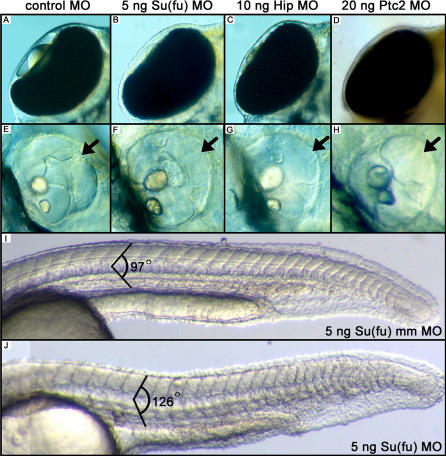
MO Injection Experiments against Su(fu), Hip, and Ptc2 (A) Dorsal view of the eye showing the lens in the eye chamber. (B–D) Dorsal view of embryos injected with the indicated MOs, resulting in a phenocopy of *dre, uki,* and *lep* mutants. (E) A wild-type ear showing the presence of the dorsolateral septum (arrow), which is not present after injections with the indicated MOs (F–H, arrow). (I) Injections with control MOs against the initiation codon of Su(fu) results in chevron-shaped somites with an angle of 97°. (J) Injection of MOs against Su(fu) results in a more obtuse angle of the somite (126°).

To identify the genes responsible for the observed phenotypes, we intended to positionally clone this class of mutants. The *dre* mutation was roughly mapped to linkage group (LG) 13 near z5395. Linkage analysis on 765 mutants reveals that the mutation was positioned close to z5395, leaving nine recombinants (0.6 cM) and z25745, leaving six recombinants (0.4 cM) (Figure 4A). Both markers mapped north of the mutation (referring to the MGH mapping panel at the Zebrafish Information Network at http://zfin.org). We identified an assembled contig of the Zv2 zebrafish genome assembly containing these markers, called ctg11890 (http://www.ensembl.org). We assumed that, based on the physical distance between these two markers, the mutation could be located on this contig. Several simple sequence length polymorphisms (SSLPs) mapping to this contig were tested for linkage, leaving zero recombinants with 11890.2A (Figure 4A). However, no marker was identified on this contig that would enclose the mutation on the south side. The closest marker was positioned in a region containing four predicted genes (Figure 4A). One of those, the β-mannosidase precursor gene, *manba,* was not considered to be a likely candidate. The other three candidates were screened for mutations by direct sequence analysis of all 28 predicted exons. This analysis revealed several silent mutations and one missense mutation in the third exon of Su(fu) [30], changing a threonine (ACG) to a lysine (AAG) at amino acid position 111 (Figure 4B), indicated as T111K. This residue is highly conserved in a stretch of eight amino acids: GFELTFRL, from bacteria *(Bacillus circulans)* to human (Figure 4C). No additional mutations affecting protein sequence could be identified in the other predicted coding regions, so we expected this mutation to be responsible for the *dre* phenotype. To test the hypothesis whether a loss of function of Su(fu) was able to phenocopy the mutants, we injected morpholino antisense oligonucleotides (MO) targeted against the predicted initiation codon of Su(fu) (Figure 5). The characteristic eye (Figure 5A and 5B) and ear (Figure 5E and 5F) phenotype of the *dre* mutant could be phenocopied effectively (75% phenocopy in two different strains, *n* = 58), in contrast to a control MO. Besides the eye and ear phenotype, the MO also induced a somite phenotype (Figure 5I and 5J). The normally chevron-shaped somites become partially flat, an effect previously described [30]. This could be due to a maternal component, which can be inhibited by the MO, enhancing the phenotype. Alternatively, the *dre^tm146d^* allele may be a partial loss of function allele. To distinguish between these possibilities, we injected up to 25 ng of MO against a splice site, thereby affecting only the zygotic component of Su(fu). This results in a clear phenocopy of the *dre* mutant without a somite defect (>90% phenocopy in two different strains, *n* = 66). Additionally, injecting the same amount of splice MO into *dre* mutants, does not enhance the phenotype (>95% phenocopy, *n* = 71), suggesting this allele of Su(fu) to be a severe loss of function.

### The *uki* and *lep* Loci Encode Negative Regulators of the Hh Signaling Pathway

**Figure 6:**
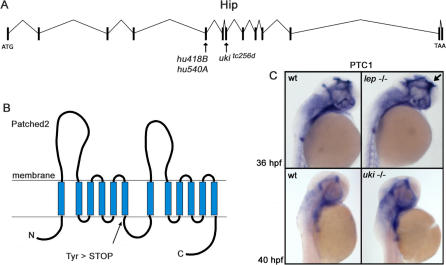
Premature Stop Codons Were Identified in the *uki* and *lep* Mutant in Hip and Ptc2, Respectively (A) Schematic representation of the genomic organization of the Hip gene. All three alleles of the *uki* mutation contain premature stop codons positioned in exon 5 and exon 7. (B) Representation of the protein structure of Ptc2 shows that the identified nonsense mutation is positioned after the sixth transmembrane domain, probably resulting in a malfunctioning protein. (C) ISH experiments show that Ptc1 expression is increased in *uki* and *lep* (arrow), confirming the aberrant activation of the Hh signaling pathway.

The similarity of phenotypes between *dre, uki,* and *lep* mutants suggested that all the mutants encode negative regulators of Hh signaling. Positional cloning of the *uki* and *lep* mutations was therefore initiated by linkage analysis of SSLPs neighboring 14 candidate genes, all members of the Hh signaling pathway. The *uki^hu418B^* mutation is tightly linked with marker z13452 and z27361 on LG 1, enclosing Hip. Sequence analysis of all predicted coding sequence of Hip revealed a nonsense mutation in exon 5, changing a tyrosine to a stop codon at position 295 (Y295STOP) of the transcript encoding 694 amino acids (Figure 6A). Sequence analysis of the *uki^hu540A^* mutant identified a stop codon in exon 5 at position 285 (Y285STOP) of the Hip protein. The *uki^tc256d^* allele contained a premature stop codon at position 406 (Y406STOP) in exon 7 (Figure 6A). Amino acids 285, 295, and 406 of the zebrafish homolog of Hip corresponds to amino acids 293, 303, and 414 in human Hip. The identified mutation in the *uki^hu418B^* mutant should lead to a truncated protein without a membrane anchor, possibly resulting in a malfunctioning protein.

Positional cloning of the *lep^tj222^* mutants was performed in a similar way. No recombination events were detected with marker z11948 and four newly identified SSLP markers on contig 10160 in genome assembly Zv2, containing Ptc2 (http://www.ensembl.org). The zebrafish Ptc2 protein has a transcript of 3,732 base pairs, encoding for 1,244 amino acids [31]. Exon sequencing identified a T to A substitution changing a tyrosine to a premature stop at position 590 (Figure 6B) directly after the sixth transmembrane domain. When this transcript is expressed, Ptc2 misses the second large extracellular domain known to be necessary for Hh binding and probably the inhibitory capability on Smo. We therefore expect this truncated protein to be a functional null.

To confirm that the identified mutations in these genes are responsible for the phenotypes, we injected wild-type embryos with an MO against the initiation codon of Hip and a splice MO for Ptc2. For the *uki* mutant, a clear phenocopy could be observed after 4 d, affecting the head, eyes, and ears (see Figure 5C and 5G) (60% phenocopy in two different strains, *n* = 64). Injection of wild-type embryos with 20 ng of Ptc2 splice MO resulted in a phenocopy of the ear and eye phenotype, however with a lower success rate (12/44; 28.5%) (see Figure 5D and 5H). This might be an effect of the positive feedback loop on Ptc2 when the Hh signaling pathway is activated, counteracting the efficiency of the MO. These experiments show that the *uki* and *lep* mutant phenotypes are indeed caused by Hip and Ptc2.

Loss of a negative regulator of Hh signaling should increase Hh activity, for which Ptc1 expression is generally used as a readout. An ISH was performed on *dre, uki,* and *lep* mutants, resulting in a slight increase in Ptc1 expression only for *uki* and *lep* mutants (Figure 6C). *dre* mutants do not show a significant increase in Ptc1 expression (data not shown).

Taken together, we conclude that aberrant activation of the Hh signaling pathway is responsible for the *uki, dre,* and *lep* mutant phenotypes.

### Double and Triple Mutants Do Not Enhance the Phenotypes

**Figure 7:**
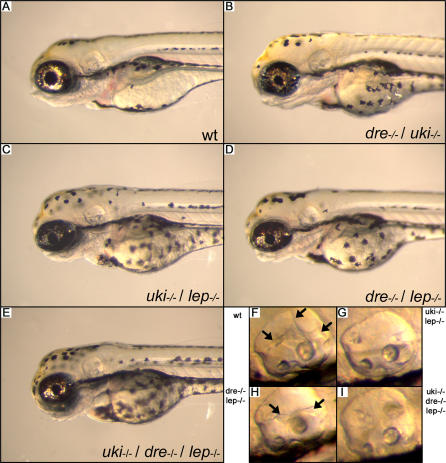
Phenotypic Analysis of *dre/uki/lep* Triple Mutants (A) Wild-type (wt) embryo at 96 hpf. (B–E) The indicated double and triple mutants do not show severe enhancement of the phenotype. (F–I) *dre/lep* double mutants have an ear phenotype comparable with a single mutant. In the *uki/lep* and *dre/uki/lep* triple mutants, the epithelial projections (arrows) fail to grow out to fuse in the middle of the ear to form the ear lumen.

Because the increase in proliferation can only significantly be detected in *uki* mutants, we initiated the generation of double and triple mutants in an attempt to enhance the level of proliferation. Current models suggest that the three genes should independently inhibit Hh signaling, therefore a higher level of proliferation could be expected. Analyzing PCNA expression in the progeny of two *uki^+/−^/dre^+/−^/lep^+/−^* carriers shows increased PCNA expression for a small subset, which upon genotyping turned out to be mainly *uki* mutants. Additionally, after sorting 96 genotyped embryos into all the possible genotypic combinations, it turned out that double and triple mutants do not show an obvious increase of the strength of the morphological phenotypes (Figure 7A–7E). The only morphological difference in double or triple mutants, compared to single mutants, comprises the ear (Figure 7F–7I). In a wild-type situation, the semicircular canal is formed after the ingrowth of the epithelial projections from the outline of the otic vesicle, which fuses in the center of the ear to form the ear lumen (Figure 7F). In the *uki/lep* double and *uki/dre/lep* triple mutants, all epithelial projections fail to grow toward the lumen of the ear (Figure 7G and 7I). These findings suggest that other negative regulators might still be present to prevent further activation of the Hh signaling pathway.

### The *uki* and *lep* Mutants Can Be Rescued by Cyclopamine Treatment

To further prove that the described mutants are a result of increased Hh signaling, we attempted to inhibit Hh signaling, and thereby rescue the mutant phenotypes, by cyclopamine treatment. Cyclopamine is an inhibitor of Hh signaling acting on the level of Smo [38] at the initial stage of the signal transduction pathway. Treating *lep* mutants with limited amounts of cyclopamine (3 μM) clearly rescued the eye and ear phenotype. Genotyping of all apparent wild-type embryos identified the presence of *lep* mutants (Table 1). Wild-type embryos were unaffected using this concentration. However, a treatment using 25 μM of cyclopamine clearly mimicked the *syu* phenotype [39], showing the functionality of the cyclopamine.

**Table 1:**
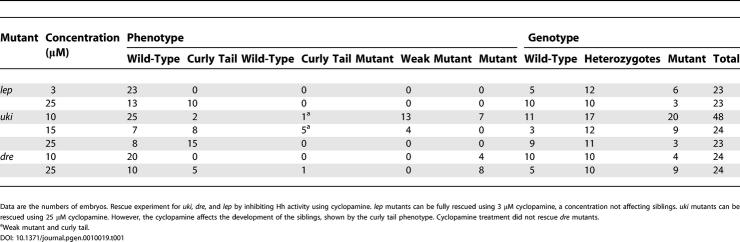
Rescuing Experiment Using Cyclopamine on *uki, dre,* and *lep* Mutants

Data are the numbers of embryos. Rescue experiment for *uki, dre,* and *lep* by inhibiting Hh activity using cyclopamine. *lep* mutants can be fully rescued using 3 μM cyclopamine, a concentration not affecting siblings. *uki* mutants can be rescued using 25 μM cyclopamine. However, the cyclopamine affects the development of the siblings, shown by the curly tail phenotype. Cyclopamine treatment did not rescue *dre* mutants.

^a^Weak mutant and curly tail.

The eye phenotype of *uki* mutant can be partially rescued using 10 or 15 μM cyclopamine, and fully rescued using 25 μM (Table 1). However, using 25 μM of cyclopamine, a subset of both siblings and mutants in the same clutch are affected, shown by a curly tail. Apparently, *uki* mutants are not protected against the effects of cyclopamine. This shows a limitation of using cyclopamine for rescuing the *uki* mutant, which is not the case for rescuing *lep* mutants, in which a much lower concentration is able to fully rescue without any side effects.

When *dre* mutants are treated with 25 μM cyclopamine, the mutant eye phenotype can be observed in combination with a curly tail, which is a result of the cyclopamine (data not shown). Increasing the cyclopamine concentration to 50 or 75 μM severely affected the development of all embryos, and therefore the eyes and ears could not be analyzed. Thus *dre* mutants are not protected against the effects of cyclopamine, but they also cannot be rescued by a cyclopamine treatment. The latter is expected because *dre*/Su(fu) acts downstream of Smo. These results emphasize that the phenotypes in this class of mutants are a result of an increased level of Hh activity.

## Discussion

### Proliferation Is Increased in the *hu418B* and *hu540A* Mutants

In our forward genetic screen, we were able to identify two proliferation mutants based on altered expression levels of PCNA and detected by an ISH approach. The identified mutants show an increase in the level of PCNA expression after 40 hpf, which is ectopically expressed in the developing tectum and in the CMZ of the eye. Unfortunately, no mutants were identified with a decreased proliferation rate. This could be explained by the fact that this screen covered only 1%–10% (100 genomes) of the zebrafish genome, leaving several additional genes to be identified in a larger screen. The limited amount of identified mutants suggests that there might be a high level of redundancy in controlling proliferation. Mutants showing an altered level of proliferation associated with increased apoptosis were excluded due to their frequent occurrence. We speculate that an increase in the amount of apoptosis results in an increase in proliferation/PCNA expression as part of a wounding/repair response [40], on which altered levels of proliferation are a secondary effect. Possibly, this secondary defect has obscured some interesting early defects.

TUNEL experiments have shown that the increase in proliferation in the *hu418B* and *hu540A* mutants is not associated with increased apoptosis. The increased PCNA expression could therefore be a result of impaired regulation of proliferation. The phenotypes observed after 4 d are similar to a previously described class of mutants identified in the large-scale Tübingen zebrafish screen [26], containing *dre, uki,* and *lep*. Complementation analysis revealed that both *hu418B* and *hu540A* are additional alleles of the *uki* mutation. *dre* and *lep* mutants are weaker than *uki* as judged from morphology and do not show a comparable increase of PCNA expression. Possibly, the increase in proliferation in the *uki* mutant might reflect a specific function for Hip, but considering the overall morphological similarity of the mutants, it is more likely that the difference is due to other factors (see below).

### Aberrant Hh Activation Is Responsible for the *dre, uki,* and *lep* Mutants

We show that *dre, uki,* and *lep* encode components of the Hh signaling pathway. The lesion in the *dre* mutant is a missense mutation in the *su(fu)* gene. The mutation, changing a threonine to a lysine, has been proposed as a potential PKC target site [41]. It is positioned in a highly conserved N-terminal region shown to be involved in binding the Gli protein [42]. Crystal structure analysis revealed that this threonine is buried and therefore suggested to be unimportant for the activity of Su(fu) [42]. However, our data indicate that this residue is crucial for the proper functioning of Su(fu), therefore it might become accessible for certain kinases due to conformational changes.

By injecting MOs against Su(fu), the mutant phenotype of *dre* could be copied, confirming that the *dre* locus encodes Su(fu). However, Su(fu) MOs against the initiation codon of Su(fu) induce a somite phenotype [30], which could be explained in a situation in which the Su(fu) MO also affects a maternal contribution. This is confirmed by the finding that an MO against a splice site does not result in a somite phenotype. Additionally, the phenotypes of *dre* can not be enhanced by the splice MO, suggesting this allele of Su(fu) to be a strong loss of function or a null. The similarity of the phenotypes within this mutant class suggested that the *uki* and *lep* mutants are also a result of aberrant activation of Hh. Linkage analysis of markers near multiple candidate genes confirmed this. Premature stop codons in Hip and Ptc2 were identified to be responsible for the *uki* and *lep* mutants, respectively, which was confirmed by the MO-induced phenocopies. In the *lep^tj222^* mutant, the identified premature stop is positioned at amino acid 590 directly after the sixth putative transmembrane domain [31], only producing the N-terminal half of the protein. In *Drosophila,* multiple alleles of Ptc have been analyzed, showing that expression of either the N- or C-terminal half alone will abolish its function [43]. We therefore expect that this severe truncation will abolish Ptc2 protein function in the mutant. The three nonsense mutations in the Hip protein result in all cases in a comparable phenotype, suggesting these alleles to be nulls. As a result, the Hh signaling pathway will be aberrantly activated, confirmed by the increase in Ptc1 expression in *uki* and *lep* mutants. However, the effect of the overactivation of the Hh pathway is subtle compared to Hh overexpression data. This might be a result of a restricted expression pattern of these negative regulators. ISH experiments showed that Su(fu) is ubiquitously expressed until 24 hpf [30] and becomes more anteriorly restricted at 42 hpf (data not shown). The expression pattern of Ptc2 is generally overlapping Ptc1 expression, with some minor differences [31]. Hip transcripts can be detected in the adaxial cells in the developing trunk and in the head, generally resembling the expression pattern of Ptc1 (Figure S1A). Within the developing trunk, two rows of adaxial cells are shown to be Hip positive at 24 hpf (Figure S1A and S1B). Hip expression is reduced in *uki* mutants at 24 hpf, which is probably due to nonsense-mediated decay (data not shown). At 40 hpf the pectoral fins, some branchial arches, and the tectum opticum are Hip positive, linking the expression pattern with the observed embryonic phenotypes (Figure S1C and S1D). Combining these expression patterns suggests that the subtle phenotypes observed in these mutants are not a result of a restricted pattern of one of these negative regulators, but are probably due to other negative regulators, most likely Ptc1, preventing further activation of the pathway.

### Phenotypic Consequences of Aberrant Hh Activation

The *uki^hu418B^* and *uki^hu540A^* mutants were picked up showing an increased level of proliferation in the developing brain. Hh activity is reported to be involved in the proliferation of cells in the central nervous system [44–49]. The increase in the volume of the head of the *uki^hu418B^* mutant is therefore in line with previous studies in which the growth of the brain is shown to be regulated by the activation of Shh-Gli1 signaling [50,51].

All the affected structures and tissues in the described mutants are known to be under control of Hh signaling during development. Hh signaling is one of the key regulators in the development of the eye, in which the formation of the retina is driven by a wave of Hh signal, secreted by the cells of the ganglion cell layer [52]. As a result, Hh controls proliferation of multiple cell types of the eye like photoreceptors and glia [53]. All described mutants show a decreased size of the pupil, which might be due to an overgrowing activity of the cells of the retina. Therefore the lens is not visible from a dorsal view, but no defects are observed in the lens itself.

The increased fin size in the *uki^hu418B^* mutant embryos could be linked to impaired Hh signaling. It is the opposite of the phenotype of *syu* mutants, in which finbuds are established, but fail to grow out, due to the absence of Shh signaling [39]. The observed phenotypes in the *dre, uki,* and *lep* mutants can therefore be linked to aberrant activation of the Hh signaling pathway.

Surprisingly, *dre, uki,* and *lep* mutants can be grown for several months, but remain small and are infertile. One explanation for the dwarfism phenotype involves the absence of growth hormone secreted by the pituitary gland. The formation of the pituitary gland is reported to be regulated by Hh signaling [54,55], indicating that its functioning might be hampered in these mutants and resulting in the observed small phenotype. However, sectioning of a *dre* mutant embryo revealed that the pituitary gland is morphologically present. The secreted hormones of the adenohypophysis are reported to be involved in the development of a dwarfism phenotype [36]. However, *dre* mutants do not show obvious altered levels of POMC, TSH, PRL, and GH expression, indicating that the formation of the adenohypophysis is not affected in this class of mutants. Currently, we are examining a potential role for the IGF signaling pathway in the development of the dwarfism phenotype.

The *dre* mutants show an abnormality in the development of the branchial arches. Normally, the primary lamellae are strictly patterned and intensely branched into secondary lamellae. Occasionally, the chondrocyte-like cells appear to form the secondary lamellae itself instead of the branching of epithelial cells. Branching of the mammalian lungs is reported to be regulated by Shh activity [56], in which increased Shh activity disrupts branching and increases the level of proliferation. The primary lamellae gain their rigidity by stacks of single chondrocytes. However, in the *dre* mutant the primary lamellae contain large clusters of prehypertrophic chondrocytes, which might be unable to start the differentiation process. The formation of cartilage is reported to be tightly regulated by the action of Indian hedgehog (Ihh) and parathyroid hormone-related protein (PTHrP) [57,58]. In this process the amount of Ihh acts like a sensor, thereby limiting the group of cells that are stimulated to enter the differentiation stage [57]. This might be deregulated in the *dre* mutant, in which an aberrant activation of Ihh signaling increases the amount of PTHrP, thereby preventing hypertrophic differentiation. The prehypertrophic chondrocytes therefore remain in their proliferative stage and might form the observed clusters in the primary lamellae. The branchial arch phenotype has not been observed in *uki/*Hip and *lep/*Ptc2 mutants, suggesting that this is a unique function for Su(fu). Therefore Su(Fu) could be modulating signals via Ptc1 as well. This is in agreement with results on Su(Fu) morphants that mimic Ptc1 morphants in their somite phenotype [30]. If the Su(fu) allele is a strong loss of function and affects signals via Ptc1 and Ptc2, why is the phenotype not any stonger? In addition to rescue by maternal protein, the role of Su(fu) in Hh inhibition may be accessory rather than absolutely central. This has been shown in *Drosophila,* in which complete inactivation of Su(Fu) does not lead to a full Hh overactivation phenotype [59].

### Complex Regulation of Hh Activity

We have shown that aberrant activation of the Hh signaling pathway is responsible for the *dre, uki,* and *lep* mutants. Nevertheless, none of the mutants that was identified shows a typical phenotype described for aberrant Hh activation as was obtained by the overexpression of dnPKA or Shh [30,60]. Surprisingly, triple mutants of *su(fu), Hip,* and *ptc2* still do not show a further increased PCNA expression or a strongly enhanced phenotype. This could be explained in a scenario in which a slight activation of the Hh pathway exceeds a certain threshold upon which Ptc1 will be expressed via an autoregulatory loop, preventing further activation of the pathway. Inhibiting Ptc1 functioning in these triple mutants could probably result in the expected Hh-overexpression phenotypes. Ptc1/Ptc2 double morphants were described, confirming this idea [30].

The ability of cyclopamine to rescue the *uki* and *lep* mutants is in line with expectations. Cyclopamine acts on the level of Smo and can revert the effect of upstream components such as Hip and Ptc2. The ability of cyclopamine to rescue the effects of mutations in Ptc is also documented for cell lines [38]. Because Su(fu) acts downstream of Smo (the point where cyclopamine acts), it is likely to be independent of the presence of an upstream signal. Similar results have been reported in a system with Gli2 overexpression, in which cyclopamine was unable to revert the effects [38]. Indeed, we find that *dre* mutants cannot be rescued by cyclopamine treatment.

Aberrant postnatal activation of the Hh signaling pathway is implicated in various types of neuronal and epithelial tumors. However, no obvious tumors have been observed in the mutants and heterozygotes. Future experiments using additional mutants, like a p53 knockout, might induce tumorigenesis in a background with aberrant Hh activity.

## Materials and Methods

### Strains and screening methods.

*uki^tc256d^, lep^tj222^,* and *dre^tm146d^* stocks were obtained from the Max Planck Institute for Developmental Biology stock center in Tübingen, Germany. ENU mutagenesis was performed on TL males as described [61]. F_1_ families were generated and interbred to obtain F_2_ families. Inbreeding generated F_3_ embryos for screening. Embryos were incubated in PTU and dechorionated by pronase treatment according to standard protocols (http://zfin.org). Staging of embryos was according to Kimmel et al. [62]. Embryos were fixed in 4% paraformaldehyde/PBS at 40 hpf. During screening, PCNA whole-mount ISH (WISH) was performed on an Abimed 96-wells ISH robot (Intavis Bioanalytical Instruments, Cologne, Germany; settings available on request). Mutants that were detected by morphological screening at 24 hpf were processed separately for WISH, along with two wild-types as controls. In addition, morphological screening was performed at 72 hpf on a duplicate clutch.

### In situ hybridization.

Manual ISH was carried out as described [63]. An antisense probe for PCNA was generated by linearizing EST clone fc43g05 (MPMGp609L0932, RZPD Deutsches Ressourcenzentrum für Genomforschung, Berlin, Germany; http://www.rzpd.de), using SalI and transcription using SP6 polymerase. Ptc1 probe synthesis was performed according to Concordet et al. [64]. ISH for POMC, TSH, GH, and PRL was performed according to Herzog et al. [65]. A 2-kb fragment of Hip was amplified from cDNA, using primers 5′-AATTTGTGCTCTTGTTAGCC-3′ and 5′-AGTGAGGTCCAGCAGGTAAG-3′, cloned and subsequently transcribed.

### TUNEL assay and BrdU labeling.

To determine the amount of apoptosis, a whole-mount TUNEL assay was performed on 40 hpf embryos as described [66]. The presence of mutants was confirmed by genotyping the analyzed embryos. BrdU labeling was performed according to a previous report [67].

### Histology.

Adult fish were fixed in 4% paraformaldehyde at 4 °C for 4 d and subsequently decalcified in 0.25M EDTA (pH 8) for 2 d. Paraffin sections (6 μm thick) were stained with eosin, Alcian Blue, or PAS in combination with hematoxylin using standard protocols.

### Measurements.

The size of the fins and pupils was determined on a Zeiss Axioplan microscope (Carl Zeiss, Jena, Germany) using a micrometer. The area of the pectoral fins was measured by determining the amount of pixels of a scanned photograph using Paint Shop Pro version 5 (Jasc Software, Corel Corporation, Ottawa, Ontario, Canada).

### Genetic mapping and positional cloning of *dre.*

The rough genome mapping of *dre^tm146d^* to LG 13 was performed by bulked segregation analysis of F_2_ embryos and genome scanning with SSLPs [68]. To fine map the mutation, a mapping strain was created by crossing a *dre/+* male in a Tübingen background to a wild-type WIK female. F_2_ fish carrying the *dre* mutation were crossed; 765 mutant F_3_ embryos were collected and genomic DNA was isolated. In total, 35 SSLPs from the Massachusetts General Hospital [69] were used for positional cloning on LG 13 in the region around 40 cM. Marker names and primer sequences can be obtained on request. Additionally, 12 SSLPs were identified on assembled contig 11890 of version Zv2 of the zebrafish genome assembly (http://www.ensembl.org) that contained the two most closely linked markers (z5395 and z25743) from the MGH database. Linkage analysis of these newly identified markers was performed to enclose the mutation. All predicted exon sequences south (corresponding to the MGH map) of the most closely linked SSLP marker were amplified and sequenced using DyeNamic ET (Amersham Biosciences, Little Chalfont, United Kingdom) protocols. All used zebrafish genome sequence data were produced by the Sanger Institute (http://www.sanger.ac.uk).

### Candidate gene approach to positional clone *uki* and *lep.*

Mapping crosses were generated crossing a female *uki^hu418B^* or *lep^tj222^* carrier in Tübingen background, with a WIK or TL male, respectively. Fourteen known regulators of the Hh signaling pathway were selected, and homologs were identified in the Zv2 zebrafish genome build at http://www.ensembl.org. A likely map position of the identified contigs was determined using the comparative map at http://zfin.org and http://www.sanger.ac.uk. Forty SSLPs surrounding these candidates were analyzed for linkage on 10 *uki* or *lep* mutants and two siblings. In the case of linkage to a candidate gene, all predicted exons were amplified and sequenced. Predicted Hip exons were obtained using EST clone fc52e12 and additional homology-based assembly of the transcript.

### MO antisense knockdown.

MOs (Gene Tools, LLC, Philomath, Oregon, United States) were designed against the predicted initiation codon or splice donor site of Su(fu), Hip, and Ptc2, along with a five-mismatch MO as a control. Their sequences are as follows (mismatches in lower case): Su(fu) MO: 5′-GCTGCTAGGCCGCATCTCATCCATC-3′, Su(fu) mismatched control: 5′-GCTGgTAcGCCGaATCTgATaCATC-3′, Su(fu) splice MO: 5′-TGACATTCTTACTCGTGAACTCTGT-3′; Hip MO: 5′-AATGCTTCATTTTTGCAGGGATGA-3′, Hip mismatched control: 5′-AATGgTTgATTTTTcCAGcGATcA-3′, Ptc2 splice MO: 5′-CTAGCAAATAAGCCATACCTGTTGT-3′, Ptc2 control 5-mm–splice MO: 5′-CTAcCAAAaAAcCCATAaCTGaTGT-3′. MOs were diluted in water to a stock concentration of 50 ng/nl. Ranges from 0.33 to 25 ng of MO were injected into one- to four-cell stage TL or ABxTL embryos and screened for the expected phenotypes 4 d after fertilization.

### Cyclopamine treatment.

Progeny from a cross of two *uki, dre,* or *lep* heterozygotes were grown in embryo medium in the presence of various concentrations of cyclopamine (from a 10 mM stock in 96% ethanol), ranging from 2 to 75 μM, administered at 5.5 hpf. Control embryos were treated with equal amounts of 96% ethanol. Genotyping was performed to identify mutants in the clutch of treated embryos.

## Supporting Information

Figure S1 Expression Pattern of Hip in Wild-Type Embryos (A and B) At 24 hpf, Hip is expressed in the brain and in two lines of adaxial cells in the developing tail.
(C and D) At 42 hpf, Hip expression is reduced in the somites. The mid-hindbrain boundary, some branchial arches, and the pectoral fins are Hip positive. Hip expression is also detected in the tectum (arrowhead).
(9.8 MB TIF)

### Accession Numbers

The GenBank (http://www.ncbi.nlm.nih.gov/Genbank) accession numbers for the described ESTs, genes, and proteins are *Bacillus circulans,* (CAD41946), zebrafish Hip EST clone fc52e12 (AI878265), PCNA EST clone fc43g05 (AI794381), *su(fu)* (NP_958466), and *ptc2* (CAB39726), and human Hip (AAH25311).

## Abbreviations

CMZ: ciliary marginal zone
ENU: ethyl-nitrosourea
GH: growth hormone
Hh: Hedgehog
hpf: hours post fertilization
Ihh: Indian hedgehog
ISH: in situ hybridization
LG: linkage group
MO: morpholino antisense oligonucleotide
PAS: Periodic Acid Schiff
PCNA: proliferating cell nuclear antigen
POMC: proopiomelanocortin
PRL: prolactin
PTHrP: parathyroid hormone-related protein
PTU: phenylthiourea
SSLP: simple sequence length polymorphism
TSH: thyroid stimulating hormone
TUNEL: terminal transferase dUTP nick-end labeling
